# Bortezomib enhances anti-tumor T cell immunity by remodeling Notch system

**DOI:** 10.1186/2051-1426-2-S3-P181

**Published:** 2014-11-06

**Authors:** Menaka Thounaojam, Duafalia Dudimah, David Carbone, Mikhail Dikov, Anil Shanker

**Affiliations:** 1 Meharry Medical College, Nashville, TN, USA; 2 Vanderbilt University, Nashville, TN, USA; 3 The Ohio State University, Columbus, OH, USA; 4 Meharry Medical College School of Medicine / Vanderbilt-Ingram Cancer Center, Nashville, TN, USA

## 

The immunosuppressive tumor microenvironment perturbs numerous immune regulatory networks and usurps host antitumor immunity. We discovered that tumor interferes with host hematopoietic Notch system in lung cancer patients. The resultant decrease in immune Notch signaling could be a major causative link in the adequate induction of antitumor immunity. Interestingly, we observed that administration of the FDA- approved proteasome inhibitor drug Bortezomib (which also sensitizes tumors to death signals) to tumor bearing mice can restore Notch signaling in lymphoid cells without increasing tumor cell proliferation or clonogenicity. Moreover, Bortezomib administration altered Notch receptor and ligand expression pattern and increased the expression of Notch target genes Hes1, Hey1 and deltex1 in thymus, lymph node and spleen. Bortezomib administration in tumor bearing mice increased IFN-g production by T cells while the proportion of regulatory T cells was decreased. Our results indicate that the activation of Hes1 and Hey1 is mediated via inhibition of NFkB pathway while deltex1 activation is mediated by PI3K pathway. In another set of experiment, we observed that administration of Bortezomib along with adoptive CD8^+ ^T cells transfer to tumor- bearing mice resulted in the reduction of tumor nodules, increased apoptosis and improve overall survival of mice. Our results clearly indicate that combining Bortezomib with adoptive T cell therapy can sustain T cell activation and function and, thus, enhances tumor immune surveillance. We are also elucidating a microRNA signature regulating immune Notch signaling. Our preliminary data suggest the role of miR-155 and miR-34a in Bortezomib induced regulation of T cell activation. The potential of Bortezomib to modulate anti-tumor Notch signaling and to enhance T cell activity presents exciting opportunities. Therapeutic restoration of immune Notch signaling by Bortezomib could help to break tumor resistance, enhance immune surveillance and sustain robust anti-tumor immunity.

